# It’s All About the IKT Approach: Three Perspectives on an Embedded Research Fellowship: Comment on "CIHR Health System Impact Fellows: Reflections on ‘Driving Change’ Within the Health System"

**DOI:** 10.15171/ijhpm.2019.31

**Published:** 2019-05-21

**Authors:** Christine E. Cassidy, Stacy Burgess, Ian D. Graham

**Affiliations:** ^1^ IWK Health Centre, Halifax, NS, Canada.; ^2^ School of Epidemiology and Public Health, University of Ottawa, Ottawa, ON, Canada.; ^3^ Centre for Practice-Changing Research, The Ottawa Hospital Research Institute, Ottawa, ON, Canada.

**Keywords:** Integrated Knowledge Translation, Co-production, Embedded Researcher, Canada, Postdoctoral Training

## Abstract

As a group of Health System Impact (HSI) postdoctoral fellows, Sim and colleagues offer their reflections on ‘driving change’ within the health system and present a framework for understanding the HSI fellow as an embedded researcher. Our commentary offers a different perspective of the fellow’s role by highlighting the integrated knowledge translation (IKT) approach we consider to be foundational to the fellowship experience. Further, we provide several recommendations to enhance Sim and colleagues’ framework to ensure we capture the full value of the fellowship program to the HSI fellow, health system organization, and academic institution.

## Introduction

In an effort to bridge the evidence to health practice/policy gap, the Canadian Institutes of Health Research (CIHR), Canada’s premier health research funding agency, established the Health System Impact (HSI) Fellowship in 2017. The goal of the fellowship program is to modernize doctoral and postdoctoral training by embedding PhD-prepared individuals and PhD trainees in a healthcare organization “to apply their research and analytic talents to critical challenges in healthcare… and to develop professional experience, new skills, and networks.”^1^ A group of HSI postdoctoral fellows from the first cohort developed a framework for understanding their experience as embedded researchers.^2^ Their framework portrays the fellow as the central agent amidst the dual health system/academic environments with the goal of ‘driving change’ in the health system. The authors also describe the fellows’ role in using an integrated knowledge translation (IKT) approach to bridge the ‘know-do gap.’ CIHR defines IKT as a collaborative model of research, where researchers and knowledge users (those in the health system setting) work together to understand and address complex healthcare problems.^3^ Each stage of the research process offers an opportunity for significant collaboration. Knowledge users bring expertise related to the relevant research topic, understanding of research findings, and are well-positioned to move these results into practice. Researchers bring methodological skills and content expertise to the partnership.^4,5^ In other jurisdictions, this general concept has been referred to by such terms as research co-production, participatory research, and engaged scholarship to name but three.^6,7^

While the framework described in their paper provides valuable insights into the trainee experience, Sim et al^2^ offer a somewhat limited interpretation of the purpose of the fellowship and the role the HSI fellow plays in supporting IKT. Collectively, as a postdoctoral fellow (CEC), health system mentor (SB), and academic mentor (IDG), we view the fellowship’s purpose with a different lens and are hesitant to conceptualize the fellows’ role as ‘driving change’ within the health system. Indeed, we would argue that expecting fellows to ‘drive’ change is unrealistic for a two-year training period and this expectation might be interpreted as hubris by those within the health system. This is not to say that the HSI fellows cannot contribute to more evidence informed decision-making within the health system that may lead to transformative change; however, the focus should be on collaborating and facilitating rather than driving change. As such, we wish to expand on Sim et al’s interpretation of the CIHR HSI fellowship and offer a different perspective on its purpose and IKT value. Our comments are intended to further this important conversation, focusing on three IKT components of the fellowship that we feel warrant further consideration and discussion, including (*i*) establishing and maintaining collaborative partnerships for research and change; (*ii*) maximizing the use of protected time for science; and (*iii*) highlighting the value added to both the academic and health system organizations (Table). We conclude by offering several recommendations we believe will enhance Sim et al’s framework for understanding the HSI fellow as an embedded researcher and the value of the IKT approach.

**Table:** Benefits of IKT Components to the HSI Fellowship Partners

	**IKT Components**		
**HSI Fellowship Partners**	**Partnerships and Collaboration**	**Maximizing the Use of Protected Time for Academic Work**	**Added Value to Health System and Academic Organizations**
HSI Fellow	Supportive environment for learning to create linkages between academia and the health system Understanding of the organization’s context by being embedded in the daily operations	Develop and hone expertise in collaborative applied health services and policy research Resources for co-production of research evidence	A pool of early career researchers who are adept at applied health services and policy research and are able to easily integrate into health system organizations or the academy Researchers who are able to bridge the worlds of research and practice/policy and contribute to reducing evidence practice/policy gaps in the health system Trusting relationships developed that lead to future collaborative projects for the fellow following the end of the PhD or postdoctoral Increased capacity within the health system for knowledge translation and evidence-informed practice/policy
Health System Organization	Greater exposure to cutting edge health services and policy research methods, including knowledge translation Having relevant research conducted that can inform the organization’s decision making Enhancing ability for evidence-informed problem-solving Creates small wins that lead to incremental change in the system	Ensures that the fellows are developing advanced research-related competencies to apply to the health system context Fellows that are effective in employing an IKT approach to research	
Academic Institution	Contributing to the collaborative training of effective and efficient health services and policy researchers Opportunity to collaborate with the fellow and health system organization on information sharing and joint problem solving Greater understanding of how the health system organization functions Production of higher quality of health services and policy evidence	Results in the next generation of researchers having excellent research and knowledge translation skills Ensures that the fellows training experiences are optimized and they acquire research skills and experiences that optimize their job market competitiveness	

Abbreviations: IKT, integrated knowledge translation; HSI, Health System Impact.

## IKT Components of the HSI Fellowship

### Partnerships and Collaboration

According to Sim et al,^2^ “the fellowship aims to bridge the knowledge-to-practice gap through propelling evidence-informed improvements in health services and health policy.” We argue that the aim of the fellowship is more than a one-way propelling of evidence into health services and policy improvements originating from the fellow. Perhaps not intended, but the language in the preceding quote does not convey the concepts of meaningful engagement and collaboration which we consider to be the cornerstone of IKT and the HSI fellowship experience. We believe the HSI fellowship offers a unique opportunity to put CIHR’s definition of IKT into action. Embedded within the fellowship structure are opportunities to build and maintain effective partnerships to support the process of change within the organization. The fellowship is designed to take IKT from a theoretical concept and create explicit partnerships from the outset to allow each team member to contribute valuable expertise. The health system mentor is expected to bring the insider perspective and detailed understanding of the complexities of the health system as well as perspective on the problems or issues that benefit from research. She or he helps the fellow to navigate the organization’s readiness for change, processes, and key players within the system. The academic mentor is expected to contribute scientific expertise and supports the fellow in conducting rigorous applied research. In our experience, the joint mentorship creates a safe environment for the fellow to learn the skills needed to build effective and trusting relationships with knowledge users throughout the organization. This collaborative structure is supporting the HSI fellow to contribute their research and analytical skills, while maintaining humility and taking time to understand the nuances of the practice or policy context. It is important for HSI fellows to stay attune to the relational components embedded within the program and not only use collaborative language to describe their role, but strive to achieve meaningful engagement and collaboration, as they are essential elements of IKT.^6,8,9^

## Maximizing the Use of Protected Time for Science

In addition to the experiential learning opportunities, the HSI fellowship structure includes advanced academic training with 30% of the fellow’s time protected for academic work. However, it is easy for the lines to blur and the time for academic work may be used for additional initiatives within the health system. It is important to highlight the value of the dedicated time for science and ensure that fellows maximize their use of this time. First, the protected time is intended to support fellows in building research-related competencies and strengthening their research and analytical skills to apply in the real-world setting. It supports the fellow to develop expertise in applied health services and policy research to be able to make important scientific contributions. Second, the explicit focus on academic work introduces the fellow to a new approach to conducting research. Instead of the traditional push of evidence from academia into the health system, the HSI fellowship is designed to provide the fellow with the skills and resources to engage in co-production of research evidence with those who will use the research. With this IKT approach, we expect the likelihood of the co-produced research findings to be of higher quality, more relevant to the organization’s context, and used in the health system.^6,10^ Lastly, we also believe there is a related and practical reason for focusing on the science as conducting rigorous research translates into more robust health system solutions.

## Added Value to the Health System and Academic Organizations

We agree with Sim et al^2^ that the opportunity to straddle both the academic and health system environments has many benefits for the HSI fellow. We would go further however and argue that the IKT components of the fellowship also have significant value for both the academic and health system organizations. From the health system perspective, it is important to temper our expectations of the “impact” fellowship and understand the value added to the organization over time. It is unrealistic to expect one fellow to drive a substantial culture shift within a 1-2 year time frame. In our experience, the fellowship supports health system partners to use an alternative approach to problem-solving and gain a stronger understanding of using evidence in their decision-making. This can create opportunities for small wins and enhanced research capacity within the organization, leading to incremental change in the system. From an academic perspective, the fellowship assists universities to support the next generation of health services researchers to become experts in navigating the complexities of the health system and working in partnership with knowledge users. Further, the academic institution may be engaged in research that is relevant to knowledge users and may be used to inform health system decision-making. Benefitting both health system and academic organizations, the fellowship is expected to build a pool of early career researchers who are adept at applied health services and policy research and are able to easily integrate into health system organizations or the academy. Lastly, from our experience, the program helps to build trusting relationships between the fellow, health system leaders, and researchers that may lead to future collaborative projects that aim to reduce evidence practice/policy gaps in the health system.

### Recommendations to Enhance Framework

We agree with Sim and colleagues that future research is needed to examine the contributions of the HSI fellowship and its impact to the fellow, health system, and academia. Building on Sim et al’s current framework,^2^ we suggest the following additions to better capture the IKT components of the fellowship and ensure they are measured to determine whether the benefits to each of the partners, as outlined in the Table, are being optimally achieved.

The framework should capture and set out to evaluate:

The impact of the IKT relationships, including how the HSI fellow, health system mentor, and academic mentor work together and how the HSI fellow partners with other members of the health system

The fellows’ contributions to the scientific community, including knowledge translation dissemination activities

The fellows’ research-related competencies and skills for IKT/co-production

The impact of the fellowship on research and knowledge translation capacity in the health system organization, from the perspective of the health system mentor and other members of the organization

The impact of the fellowship on decision-making in the health system organization (what we referred to above as ‘small wins’ in the health system)

The impact of the fellowship on the fellows’ career goals and career trajectory

It is important that this evaluation does not rely solely on a set of counting metrics to measure the fellows’ impact and success (ie, number of peer reviewed publications, number of engagement and training events, etc). Innovative approaches should be considered to examine how the fellows, academic researchers, and health system decision-makers work together to achieve their impact goals. This would contribute to our understanding of the process and culture of co-producing research to improve health and health system outcomes.

## Conclusion

Our joint interpretation of the CIHR HSI fellowship is relevant and timely for moving the conversation around IKT forward. By outlining three IKT components of the fellowship, we have highlighted the potential value of this innovative program to the PhD/postdoctoral fellow, health system organization, and academic institution. We recommend building on Sim et al’s framework to ensure we are clearly describing the role of the HSI fellow and capturing the full value of the fellowship to all team members. As the HSI fellowship program continues to grow and modernize doctoral and postdoctoral training, we anticipate more IKT partnerships and co-production efforts between academia and the health system. Ideally, this will lead to more relevant research findings and contribute to reducing evidence practice/policy gaps in the health system.

## Acknowledgements

CEC’s Health System Impact Fellowship is co-funded by the CIHR-Institute of Health Services and Policy Research, Nova Scotia Health Research Foundation, and IWK Health Centre. IDG is a recipient of a CIHR Foundation Grant – the Integrated Knowledge Translation Research Network (IKTRN) (FDN #143237). CEC is a trainee of the IKTRN.

IDG was the chair of the 2018 CIHR Health System Impact Fellowship (Doctoral Stream) peer review committee. For the 2019 CIHR Health System Impact Fellowship peer review meetings, IDG will be the chair of the postdoctoral stream committee and scientific officer of the doctoral stream committee.

## Ethical issues

Not applicable.

## Competing interests

Authors declare that they have no competing interests.

## Authors’ contributions

All authors conceptualized the manuscript. CEC prepared the initial manuscript draft. SB and IDG provided critical revision of the manuscript. All authors read and approved the final manuscript.

## Authors’ affiliations

^1^IWK Health Centre, Halifax, NS, Canada. ^2^School of Epidemiology and Public Health, University of Ottawa, Ottawa, ON, Canada. ^3^Centre for Practice-Changing Research, The Ottawa Hospital Research Institute, Ottawa, ON, Canada.
